# Delayed Re-epithelialization After Epithelium-Off Crosslinking: Predictors and Impact on Keratoconus Progression

**DOI:** 10.3389/fmed.2021.657993

**Published:** 2021-10-15

**Authors:** Chiara Bonzano, Carlo Alberto Cutolo, Donatella Musetti, Ilaria Di Mola, Chiara Pizzorno, Riccardo Scotto, Carlo Enrico Traverso

**Affiliations:** Eye Clinic, DiNOGMI, University of Genoa and IRCCS San Martino Polyclinic Hospital, Genoa, Italy

**Keywords:** epithelium-off crosslinking, accelerated corneal crosslinking, keratoconus, delayed re-epithelialization, bandage contact lens

## Abstract

**Purpose:** To investigate the demographic and corneal factors associated with the occurrence of delayed reepithelialization (DRE) after epithelium-off crosslinking (epi-off CXL).

**Design:**Retrospective case series.

**Methods:**A chart review was performed to identify patients treated with epi-off CXL. DRE was defined as a corneal epithelial defect detected by fluorescein staining that persisted for more than 10 days. Slit-lamp examination, anterior segment optical coherence tomography, corneal topography, and corneal *in vivo* confocal microscopy (IVCM) were always performed preoperatively and at each follow-up visit (1, 3, 6, 12 months). A generalized estimating equation was used to assess the baseline factors associated with DRE.

**Results:**Data from 153 eyes were analyzed. The mean age of patients was 24.9 ± 8.5 years, and 47 (30.7%) were women. The average reepithelization time was 4.7 ± 1.8 days. Six eyes (3.9%) experienced DRE. In the multivariate model, both the age of the patient (OR = 1.30; *p* = 0.02) and the corneal steepest meridian (OR = 0.44, *p* = 0.047) were associated with DRE. Baseline nerve count was also associated with DRE (0.87, *p* = 0.03). Male gender was associated with a slower early nerve regrowth (1–6 months) (*p* = 0.048), but not with the occurrence of DRE (*p* = 0.27). Preoperative central corneal thickness was not related to DRE (*p* = 0.16). DRE was not associated with keratoconus progression after epi-off CXL (*p* = 0.520).

**Conclusions:**The association between DRE and age may reflect the age-related decrease in the corneal healing response. Also, low baseline corneal nerve count is associated with DRE. Gender seems to affect reinnervation measured by IVCM but not the reepithelization time. DRE does not seem to affect the efficacy of epi-off CXL.

## Introduction

Keratoconus (KC) is a non-inflammatory corneal disease characterized by progressive ectasia, in which stromal thinning and cornea weakening can lead to an increase of anterior and posterior corneal curvature (1). The resulting irregular astigmatism, myopia together with the progressive corneal scarring, is responsible for visual loss (2).

Corneal collagen crosslinking (CXL) is a parasurgical technique of corneal tissue strengthening. Riboflavin activated by irradiation with ultraviolet-A (UVA) light increases the intra and interfibrillar covalent bonds, thereby increasing the mechanical strength and slowing the progression of corneal ectasia (3).

The epithelium-off CXL (epi-off CXL) technique is deemed a safe procedure for the treatment of progressive KC (4–8).

To reduce discomfort and to promote fast and safe epithelial healing, patients are generally given daily topical antibiotics and corticosteroids for 1 to 2 weeks following the epi-off CXL with close follow-up. A bandage contact lens is usually placed following the procedure, night and day up to 3–5 consecutive days. Three days later, after lens removal, complete epithelial healing is observed at the biomicroscopic examination in most of the cases (9).

Delayed reepithelialization (DRE), defined as a corneal epithelial defect detected by fluorescein-staining that persisted for more than 10 days after treatment, is a possible complication of epi-off CXL.

This study aims to investigate the demographic and corneal factors associated with DRE.

## Patients and Methods

A retrospective chart review was performed to identify patients treated with epi-off CXL at ClinicaOculistica, University of Genova, Italy. All subjects provided written informed consent.

Inclusion criteria were as follows: patients affected by grade II–III KC (Amsler-Krumeich (AK) grading); clinical and instrumental progression documented by repeated corneal topography over at least 6 months intended as an increase in the steep meridian value (*K*_max_) of 1.0 diopter or more; and willingness to undergo epi-off CXL.

Diagnosis of KC was established by using the AK classification, based on spectacle refraction, central keratometry, corneal transparency, and corneal thickness.

We included eyes with early to moderate progressive KC, corneal thickness >400 μm, and with minimum of 12 months follow-up after epi-off CXL.

Exclusion criteria were advanced KC with stromal scarring, corneal hydrops, herpetic keratitis, autoimmune and other systemic diseases, pregnancy, and breastfeeding.

### Patient Assessment

Slit-lamp examination implemented with corneal epithelial fluorescein staining, anterior segment optical coherence tomography (AS-OCT) (RTVue, Optovue Inc., Fremont, CA), corneal topography measurement using TMS-4 topographer (Tomey Corporation, Tokyo, Japan) with surface regularity index, and corneal *in vivo* confocal microscopy (IVCM) (Heidelberg Retina Tomograph II, Rostock Cornea Module) were always performed preoperatively and at each follow-up visit (1, 3, 6, and 12 months).

### Surgical Technique

Epi-off CXL was always performed by the same surgeon (C.B.) using the accelerated protocol that uses equivalent total irradiance [9 mW/cm for 10 min, 5.4 J/cm (A9/10-CXL)] (10).

The procedure was always performed under sterile operating conditions using topical anesthesia oxybuprocaine hydrochloride 0.4% (Alfa Intes—Ind.Ter.Splendore) anesthetic drops. Topical pilocarpine 2.0% was administered 20 min before treatment.

After the application of an eyelid speculum, epithelial removal (9-mm) was achieved using a blunt knife. Riboflavin (0.1% in 20% dextran solution; Ricrolin; Sooft, Montegiorgio, Italy) was administered topically every minute for 15 min. The administration was continued every 2 min during UVA exposure.

The UVA irradiation was performed with a CBM X-Linker Vega using a 9 mW/cm^2^ to obtain 10 min of UVA irradiation on balance while delivering a standard energy dose of 5.4 J/cm^2^.

The post-CXL medication consisted of antibiotic eye drops solution (Netilmicin 0.3%) (3mg/ml) (four times daily for 1 week) and dexamethasone sodium phosphate (0.1%) (1mg/ml) (four times daily for 1 week and tapered over the following 7 days). Preservative-free isotonic solution (hyaluronic acid (HA) 0.4% and taurine (TAU) 0.5%) and preservative-free B2 vitamin eye drops (Ribolisin free, SOOFT italia) were used for 4 weeks. Oral pain medications (Tramadol 50 mg, 1–2 per day; diclofenac 25 mg, 1–2 per a day) were prescribed on the treatment day and the day after. A specific bandage lens for injured tissues, with a regenerating, anti-inflammatory, and analgesic effect (Regenera Therapeutic Lens) (16,5 mm, hydrogel Filcon II 3 e 75% H_2_O, Dk = 42) was placed after the procedure to reduce the discomfort and to promote the epithelial healing. It was removed after 3 days if the epithelial healing was complete.

Delayed reepithelialization was defined as a corneal epithelial defect detected by fluorescein staining at the slit lamp examination that persisted for more than 10 days after epi-off CXL.

Delayed reepithelialization was managed conservatively with topical medication and bandage contact lens that was replaced every 3 days until complete healing. In one case, debridement was performed for redundant or loose epithelial margins. Additional surgical procedures were not needed in our cohort of patients (11, 12).

### Statistical Analysis

Data are reported as mean (standard deviation) for continuous parameters or as frequencies for categorical parameters.

A generalized estimating equation was used to assess the baseline factors associated with DRE and to account for the correlation between fellow eyes. DRE was considered as the dependent variable in the analysis. Then a multivariate model was built. Criteria for model selection were guided by the univariate analysis and clinical significance of the variables. Univariate linear regression was also used to assess the association between baseline characteristics and the speed of nerve regrowth between 1 and 6 months. All statistical analyses were performed with Stata version 15.1 (StataCorp LP, College Station, TX). The alpha level (type I error) was set at 0.05 for all analyses.

## Results

**Figure 1:**
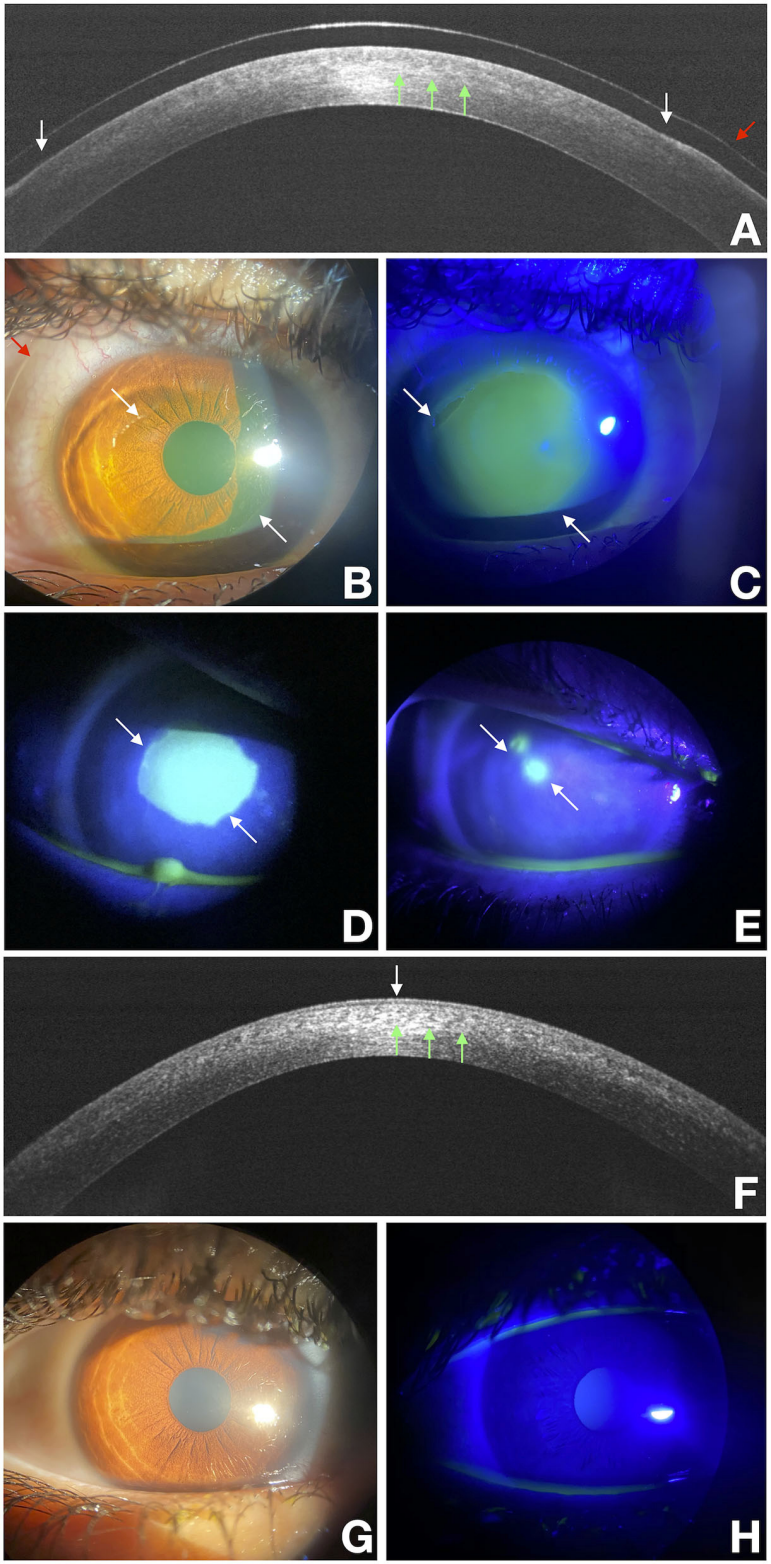
Delayed re-epithelialization after the epithelium-off CXL (epi-off CXL) imaged with a multimodal approach. White arrows point at the edges of the epithelial fronts and green arrows points to the demarcation line, and the red arrow points to the bandage lens. OCT scan over the disepithelized area at baseline **(A)**. Photograph obtained at baseline with diffuse white light **(B)** and with fluorescein staining (green) photograph obtained under cobalt-blue light illumination **(C)** imaged at baseline. **(D,E)** were acquired at the end of treatment at 3 and 10 days respectively. They show a residual corneal epithelial disepithelialization. OCT scan over the re-epithelialization area at day 15 **(F)**. Photograph obtained at day 15 with diffuse white light **(G)** and with fluorescein staining (green) photograph obtained under cobalt-blue light illumination **(H)**.

Based on inclusion and exclusion criteria, we analyzed data from 153 patients. The average reepithelization time was 4.7 ± 1.8 days (SD). Postoperative corneal biomicroscopic examination performed on the third day after treatment showed a clear cornea, little edema, and no opacities before and immediately after therapeutic contact lens removal. Seventy-two hours after epithelium removal, almost all the patients had complete reepithelialization as shown by the fluorescein dye test instilled in the eye, only six eyes (3.9%) experienced DRE (Figure 1). Among these no one reported either a corneal infection or KC progression after epi-off CXL. Patient demographics and baseline ocular characteristics of the two groups are summarized in Table 1. The mean age of patients was 24.5 ± 8.3 in patients who did not experience DRE, and 33.6 ± 7.5 in patients with DRE (*p* = 0.01). As regard ocular characteristics, nerve count was 70.4 ± 19.8 and 36.5 ± 4.9 in the patient without and with DRE (*p* = 0.01), respectively. No significant differences were observed between the two groups regarding other demographic or ocular characteristics. As described in the methods, a model was built to better identify ocular and demographic characteristics associated with DRE (Table 2). The age of the patient and corneal nerve count were associated with DRE in univariate analysis with OR = 1.11 (*p* = 0.02) and OR = 0.89 (*p* = 0.01), respectively. Then, we have built two different multivariate models, not including nerve count (model 1) or age (model 2), to avoid multicollinearity. In both the multivariate models, age and nerve count remained significantly associated with DRE. Nerve count and age were also found negatively correlated (r = −0.27; *p* = 0.028). In model 1 the steepest meridian value was associated with DRE whereas in model 2 this variable was only marginally associated with DRE. Then, the same variables were tested for association with nerve regrowth (1–6 months), and it was found that the male gender was the only variable significantly associated with a slower early nerve regrowth (*p* = 0.048). Of note, even if gender was included in the multivariate models, it did not associate with DRE (*p* = 0.27 model 1). We also tested the hypothesis that DRE could affect the efficacy of epi-off CXL, and we found that DRE was not associated with KC progression after treatment (*p* = 0.520).

**Table 1:** Demographic and clinical details for patients that experienced.

	**No DRE (*N* = 147)**	**DRE (*N* = 6)**	***p*** **-value**
Age, (mean ± sd) years old	24.5 ± 8.3	33.6 ± 7.5	0.01
Gender, female	45 (30.6%)	2 (33%)	0.88
K2,(mean ± sd), diopters	44.2 ± 2.2	45.9 ± 3.7	0.31
CCT at thinnest point, (mean ± sd), μm	497 ± 34	479 ± 19.4	0.20
CCTmin, (mean ± sd), μm	463 ± 35	442 ± 11	0.16
GAT IOP, (mean ± sd), mmHg	13.0 ± 2.0	11.5 ± 1.7	0.14
BCVA, (mean ± sd), decimals	6.8 ± 2.5	8.3 ± 1.9	0.14
SF, (mean ± sd), diopters	−1.2 ± 2.8	−0.38 ± 1.59	0.46
CYL, (mean ± sd), diopters	5.6± 2.9	4.2 ± 1.3	0.30
SRI (mean ± sd)	1.02 ± 0.37	0.85 ± 0.15	0.36
Nerve, (mean ± sd), n	70.4 ± 19.8	36.5 ± 4.9	0.01
Dendritic cells, (mean ± sd), n	17.1 ± 15.6	16.0 ± 4.2	0.91

*DRE, Delayed re-epithelialization; K2, steepest meridian reading; CCT, central corneal thickness; IOP, intraocular pression measured with Goldmann tonometry; BVCA, best corrected visual acuity; SF, sphere; CYL, Cylinder; SRI, surface regularity index*.

**Table 2:** Analysis of the association between baseline demographic and anatomical parameters with DRE.

	**Univariate**			**Multivariate (model 1)**			**Multivariate (model 2)**		
	**OR**	**95% CI**	**p**	**OR**	**95% CI**	***p***	**OR**	**95% CI**	**p**
Age (years)	1.11	1.01–1.20	0.02	1.30	1.04–1.62	0.019			
Female	1.13	0.20–6.41	0.89	0.24	0.02–3.00	0.270	0.37	0.02–9.16	0.547
Steepest meridian (diopters)	0.84	0.59–1.17	0.26	0.44	0.19–0.99	0.047	0.61	0.34–1.09	0.099
Cylinder (diopters)	0.80	0.52–1.22	0.25						
CCT (μm)	0.98	0.96–1.01	0.20						
CCT at thinnest point (μm)	0.98	0.96–1.01	0.16	0.95	0.91–1.00	0.066	0.96	0.91–1.02	0.147
IOP (mmHg)	0.71	0.44–1.13	0.15						
BCVA (decimals)	1.35	0.89–2.03	0.15						
Sphere (diopter)	1.24	0.71–2.14	0.45						
SRI	0.34	0.03–3.50	0.35						
Nerve	0.89	0.81–0.97	0.01				0.87	0.76–0.98	0.026
Dendritic cells	0.99	0.90–1.10	0.92						

*OR, Odds ratio; CCT, central corneal thickness; IOP, intraocular pression measured with Goldmann tonometry; BVCA, best corrected visual acuity; SRI, surface regularity index*.

## Discussion

The association between DRE and age may reflect the age-related decrease in the corneal healing response. Gipson et al. (13) and some other studies reported that corneal wound healing declines with age (13–16). Major well-known changes in the cornea with age include the thickening of both the epithelial and endothelial basement membranes.

By regulating the growth factor activity, the basement membrane plays a key role in the cellular reparative process (17). Its hemidesmosome-anchoring fibrils bind the basal cells membrane to the Bowman's layer and form anchoring complexes by binding to the stromal plaques (18). The anchoring fibrils seem to become disrupted with increasing age, and the membrane thickness exceeds fibril length, and it could effectively block linkage between the anchoring fibrils and Bowman's layer (19). Furthermore, there is a well-known diminution of sex hormones that occurs with age in both sexes that affect the glandular functions and compromise the ocular surface system, and consecutively the cascade of healing mechanisms (20). Besides, the number of nerves in the corneal epithelial subbasal plexus decreases with age, leading perhaps to the loss of sensitivity observed with age involving at first the corneal periphery and successively spreading toward the central zone (16, 21). We have to keep in mind that the corneal sensation has already nearly disappeared in the early post epi-off CXL period, it improved to its baseline levels only at sixth postoperative month according to Ozgurhan et al. (22). The lower the corneal sensitivity, the lower the trend of the corneal epithelium to heal. In our analysis, we showed that baseline nerve count seems to play a role in the corneal healing process. Last but not least, an aging-related decrease in the number of conjunctival keratocytes has been reported (23). It could mean a lower level of Muc16, conjunctival mucin, which affects the behaviors of the corneal epithelium and keratocytes (24).

Gender seems to affect reinnervation measured by IVCM but not the reepithelization time. Up to now, different studies stated that gender does not have any influence on reepithelialization as in our experience (25). Instead, no reports in literature found any impact of gender on the corneal reinnervation, unlike our observation. This study is limited by the small number of eyes who experienced complications after CXL and DRE. Even if CXL is a safe procedure, it is clinically meaningful to identify patients at risk for DRE.

The association between corneal steepest meridian readings and DRE is an interesting issue. It has been pointed out that the epithelium at the cone apex is thinner, where the stroma is steeper.

An overall thinning of the epithelium across the ectatic cornea and an apparent difference in epithelial thickness, which is lower in the central region and higher toward the inferior keratoconic cornea, is observed. Such irregularity could explain a slower reepithelialization. Vinciguerra et al. reported that the epithelium could act as a smoothing agent that reduces corneal power, astigmatism, and cornea irregularity after epi-off CXL (26). The reepithelialization and the following remodeling effect of CXL can take about 6 months to flatten and regularize the keratoconic shape of the cornea (26). This slower epithelium remodeling process when the conus is steeper could explain why topography obtained 1 month after CXL paradoxically shows an increase in the steepness of the cone.

The present study suggests that patients who experienced DRE did not derive less efficacy from epi-off CXL.

## Conclusion

The association between DRE and age may reflect the age-related decrease in the corneal healing response. Also, low baseline corneal nerve count is associated with DRE. Gender seems to affect reinnervation measured by IVCM, but not the reepithelization time. DRE does not seem to affect the efficacy of epi-off CXL treatment.

## Data Availability Statement

The raw data supporting the conclusions of this article will be made available by the authors, without undue reservation.

## Ethics Statement

Ethical review and approval was not required for the study on human participants in accordance with the local legislation and institutional requirements. Written informed consent to participate in this study was provided by the participants' legal guardian/next of kin.

## Author Contributions

CB: conceptualization, methodology, investigation, writing, and editing the final manuscript. CC: conceptualization, methodology, formal analysis, and editing the final manuscript. DM, RS, ID, and CP: resources and data curation. CT: supervision and reviewing.

## Conflict of Interest

The authors declare that the research was conducted in the absence of any commercial or financial relationships that could be construed as a potential conflict of interest.

## Publisher's Note

All claims expressed in this article are solely those of the authors and do not necessarily represent those of their affiliated organizations, or those of the publisher, the editors and the reviewers. Any product that may be evaluated in this article, or claim that may be made by its manufacturer, is not guaranteed or endorsed by the publisher.

## References

[1] Sharif R Bak-Nielsen S Hjortdal J Karamichos D. Pathogenesis of keratoconus: the intriguing therapeutic potential of prolactin-inducible protein. Prog Retin Eye Res. (2018) 67:150–67. 10.1016/j.preteyeres.2018.05.002 29758268 PMC6235698

[2] Rabinowitz YS. Keratoconus. Surv Ophthalmol. (1998) 42:297–319. 10.1016/S0039-6257(97)00119-7 9493273

[3] Wollensak G Spoerl E Seiler T. Riboflavin/ultraviolet-a-induced collagen crosslinking for the treatment of keratoconus. Am J Ophthalmol. (2003) 135:620–7. 10.1016/S0002-9394(02)02220-1 12719068

[4] Mazzotta C Baiocchi S Bagaglia SA Fruschelli M Meduri A Rechichi M. Accelerated 15 mW pulsed-light crosslinking to treat progressive keratoconus: two-year clinical results. J Cataract Refract Surg. (2017) 43:1081–8. 10.1016/j.jcrs.2017.05.030 28917411

[5] Wittig-Silva C Chan E Islam FMA Wu T Whiting M Snibson GR. Randomized, controlled trial of corneal collagen cross-linking in progressive keratoconus. Ophthalmology. (2014) 121:812–21. 10.1016/j.ophtha.2013.10.028 24393351

[6] Raiskup F Theuring A Pillunat LE Spoerl E. Corneal collagen crosslinking with riboflavin and ultraviolet-A light in progressive keratoconus: ten-year results. J Cataract Refract Surg. (2015) 41:41–6. 10.1016/j.jcrs.2014.09.033 25532633

[7] O'Brart DPS Patel P Lascaratos G. Corneal cross-linking to halt the progression of keratoconus and corneal ectasia: seven-year follow-up. Am J Ophthalmol. (2015) 160:1154–63. 10.1016/j.ajo.2015.08.023 26307513

[8] Raiskup-Wolf F Hoyer A Spoerl E Pillunat LE. Collagen crosslinking with riboflavin and ultraviolet-A light in keratoconus: long-term results. J Cataract Refract Surg. (2008) 34:796–801. 10.1016/j.jcrs.2007.12.039 18471635

[9] Soeters N Wisse RPL Godefrooij DA Imhof SM Tahzib NG. Transepithelial versus epithelium-off corneal cross-linking for the treatment of progressive keratoconus: a randomized controlled trial. Am J Ophthalmol. (2015) 159:821–828.e3. 10.1016/j.ajo.2015.02.005 25703475

[10] Lang PZ. HafeziNL, Khandelwal SS, Torres-Netto EA, Hafezi F, Randleman JB. Comparative functional outcomes after corneal crosslinking using standard, accelerated, and accelerated with higher total fluence protocols. Cornea. (2019) 38:433–41. 10.1097/ICO.0000000000001878 30681515

[11] Krysik K Dobrowolski D Wylegała EA Lyssek-Boroń A Amniotic membrane as a main component in treatment supporting healing and patch grafts in corneal melting and perforations. J Ophthalol. (2020) 4238919:1–7. 10.1155/2020/4238919 32148944 PMC7042504

[12] Leccisotti A Mitomycin C. in photorefractive keratectomy: effect on epithelialization and predictability. Cornea. (2008) 27:288–91. 10.1097/ICO.0b013e31815c5a51 18362654

[13] Gipson IK. Age-related changes and diseases of the ocular surface and cornea. Investigat Opthalmol Visual Sci. (2013) 54:ORSF48. 10.1167/iovs.13-12840 24335068

[14] Faragher RG Mulholland B Tuft SJ Sandeman S Khaw PT. Aging and the cornea. Br J Ophthalmol. (1997) 81:814–7. 10.1136/bjo.81.10.814 9486017 PMC1722015

[15] Rao SN Chuck RS Chang AH LaBree L McDonnell PJ. Effect of age on the refractive outcome of myopic photorefractive keratectomy. J Cataract Refract Surg. (2000) 26:543–6. 10.1016/S0886-3350(99)00465-4 10771227

[16] Niederer RL Perumal D Sherwin T McGhee CNJ. Age-related differences in the normal human cornea: a laser scanning in vivo confocal microscopy study. Br J Ophthalmol. (2007) 91:1165–9. 10.1136/bjo.2006.112656 17389741 PMC1954900

[17] Torricelli AAM Singh V Santhiago MR Wilson SE. The Corneal epithelial basement membrane: structure, function, and disease. Investigat Opthalmol Visual Sci. (2013) 54:6390. 10.1167/iovs.13-12547 24078382 PMC3787659

[18] Mantelli F Mauris J Argüeso P. The ocular surface epithelial barrier and other mechanisms of mucosal protection: from allergy to infectious diseases. Curr Opin Allergy Clin Immunol. (2013) 13:563–8. 10.1097/ACI.0b013e3283645899 23974687 PMC3858173

[19] Alvarado J Murphy C Juster R. Age-related changes in the basement membrane of the human corneal epithelium. Invest Ophthalmol Vis Sci. (1983) 24:1015–28. 6874267

[20] Knop E Knop N Millar T Obata H Sullivan DA. The International workshop on meibomian gland dysfunction: report of the subcommittee on anatomy, physiology, and pathophysiology of the meibomian gland. Investigat Opthalmol Visual Sci. (2011) 52:1938. 10.1167/iovs.10-6997c 21450915 PMC3072159

[21] Roszkowska AM Colosi P Ferreri FMB Galasso S. Age-related modifications of corneal sensitivity. Ophthalmologica. (2004) 218:350–5. 10.1159/000079478 15334017

[22] Ozgurhan EB Celik U Bozkurt E Demirok A. Evaluation of subbasal nerve morphology and corneal sensation after accelerated corneal collagen cross-linking treatment on keratoconus. Curr Eye Res. (2015) 40:484–9. 10.3109/02713683.2014.932387 24979260

[23] Berlau J Becker H-H Stave J Oriwol C Guthoff RF. Depth and age-dependent distribution of keratocytes in healthy human corneas: a study using scanning-slit confocal microscopy *in vivo*. J Cataract Refract Surg. (2002) 28:611–6. 10.1016/S0886-3350(01)01227-5 11955900

[24] Shirai K Okada Y Cheon D-J. Effects of the loss of conjunctival Muc16 on corneal epithelium and stroma in mice. Investigat Opthalmol Vis Sci. (2014) 55:3626. 10.1167/iovs.13-12955 24812549 PMC4581617

[25] Pastar I Stojadinovic O Yin NC. Epithelialization in wound healing: a comprehensive review. Adv Wound Care. (2014) 3:445–64. 10.1089/wound.2013.0473 25032064 PMC4086220

[26] Vinciguerra P. Intraoperative and postoperative effects of corneal collagen cross-linking on progressive keratoconus. Archiv Ophthalmol. (2009) 127:1258. 10.1001/archophthalmol.2009.205 19822840

